# Impact of Poverty on Health in Developing Countries

**DOI:** 10.1002/puh2.70314

**Published:** 2026-07-16

**Authors:** Xiaoxiao Yu, Tajul Ariffin Masron, Dansha Zhang, Tutik Wiryanti Gondo

**Affiliations:** ^1^ Universiti Sains Malaysia Minden Malaysia; ^2^ Nanning College for Vocational Technology Nanning China; ^3^ Universitas Widya Mataram Yogyakarta Indonesia

**Keywords:** developing countries, generalized method of moments (GMM), health outcomes, poverty

## Abstract

This study examines the relationship between poverty and health outcomes in 63 developing countries using four available observation years within the 2013–2022 period. Using a dynamic panel framework with difference and system generalized method of moments (GMM) estimations, the analysis accounts for economic, environmental, and policy factors, while addressing persistence and endogeneity. The results indicate a generally negative association between poverty and population health, particularly in the difference GMM estimations. The effects are more pronounced in basic dimensions such as nutrition, whereas outcomes linked to institutional access, such as medical services, appear less responsive. A threshold pattern is also observed, with stronger impacts at lower and moderate poverty levels. These findings suggest that economic growth alone does not necessarily translate into health improvements, pointing to the importance of direct poverty reduction and investments in basic living conditions.

## Graphical abstract

This study found that poverty is a key constraint on health in developing countries, and that poverty reduction in low‐ to moderate‐level poverty has a more significant marginal effect on health improvement, far exceeding the impact of economic growth alone.

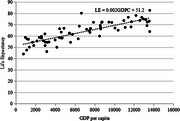

## 1 Introduction

Health is widely recognized as a fundamental indicator of social welfare and economic development. Improvements in population health contribute not only to individual well‐being but also to productivity, human capital accumulation, and long‐term economic growth [1, 2]. A large body of literature has documented that economic conditions such as income, education, and public investment play a critical role in shaping health outcomes. For example, higher income levels are generally associated with longer life expectancy and lower mortality rates [3], whereas education enhances health awareness and access to healthcare services. However, despite sustained economic growth in many developing countries, improvements in health outcomes have been uneven and, in some cases, limited. Several low‐ and middle‐income countries continue to experience high mortality rates, malnutrition, and inadequate access to healthcare services, even as macroeconomic indicators such as gross domestic product (GDP) per capita improve. This suggests that economic growth alone may not be sufficient to ensure better health outcomes, particularly in contexts characterized by persistent poverty and structural constraints [2].

To illustrate this issue, Table 1 presents summary statistics of key economic and health indicators across 63 developing countries. The data reveal substantial variation in health outcomes despite comparable levels of economic development. In particular, whereas GDP per capita shows moderate dispersion, health indicators such as life expectancy and mortality exhibit wide variability, suggesting that income alone does not determine health outcomes.

**TABLE 1:** Summary of economic and health indicators of 63 developing countries.

Variable	Mean	Min	Max	Std. Dev.
GDP per capita ($)	3816	450	13,626	3180
Life expectancy (years)	72.06	32.19	98.09	13.29
Poverty gap (%)	∼20	∼2	∼60	High variation
Mortality rate (per 1000)	High variation	—	—	—

Abbreviation: GDP, gross domestic product.

*Source:* References [4, 5].

**FIGURE 1:**
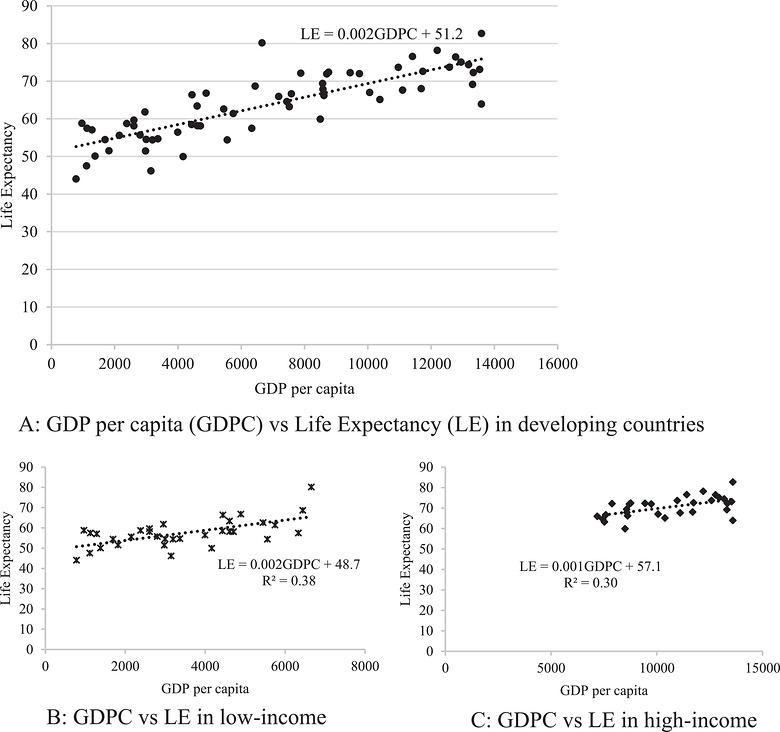
(A) GDP per capita (GDPC) versus life expectancy (LE) in developing countries. (B) GDPC versus LE in low‐income. (C) GDPC versus LE in high‐income. The equation represents the regression line. *Source:* References [4, 5].

Figure 1 illustrates the relationship between GDP per capita and life expectancy across developing countries. Although a positive association is observed, the relationship is weak and highly dispersed (Figure 1A), indicating that higher income does not consistently translate into better health outcomes. Countries with similar income levels exhibit substantial variation in life expectancy, suggesting that additional structural factors play an important role. Moreover, the relationship is stronger in low‐income countries (Figure 1B) but weakens at higher income levels (Figure 1C), reflecting diminishing returns of income on health. These patterns imply that poverty‐related constraints, such as limited access to healthcare, inadequate living conditions, and environmental risks, continue to shape health outcomes beyond income alone.

A particularly important dimension of this issue is the rise of urban poverty in developing countries. Rapid urbanization has increased income opportunities but also intensified exposure to environmental risks, such as air pollution, overcrowding, and urban heat stress. Evidence shows that urban populations in low‐ and middle‐income countries are increasingly exposed to hazardous levels of air pollution and extreme heat, both of which are associated with adverse health outcomes [6, 7]. In such contexts, higher income does not necessarily translate into better health when environmental and infrastructural conditions are inadequate. This “urban health paradox” suggests that the relationship between income and health is conditional rather than universal. Economic gains may be offset by environmental degradation, inequality, and limited access to quality public services. As a result, focusing solely on income‐based explanations risks overlooking key mechanisms that shape health outcomes in developing countries.

At the same time, recent studies have emphasized that the relationship between economic conditions and health is complex and multidimensional. Economic growth may improve health through increased income and better access to services, but it may also generate environmental pressures or exacerbate inequalities that offset these gains [8, 9]. These findings suggest that improvements in economic conditions do not uniformly translate into better health outcomes, particularly in the presence of persistent poverty. In such contexts, poverty remains closely associated with limited access to healthcare, inadequate nutrition, and poor living conditions, which can constrain overall health outcomes despite economic progress.

Despite this growing recognition, the existing literature remains fragmented in several important ways. First, many empirical studies focus on individual determinants such as income, education, or health expenditure without examining their combined influence within a unified empirical framework [3, 10, 11]. Second, there is limited cross‐country evidence that integrates poverty, economic conditions, environmental factors, and health strategies (HSs) within a unified empirical framework [2, 7, 9]. Third, although health outcomes are inherently persistent over time, relatively few studies explicitly account for this dynamic structure in a multi‐country setting using appropriate econometric approaches [12, 13, 14]. Furthermore, the role of poverty as a structural constraint on health outcomes has not been sufficiently examined in an integrated empirical framework. Poverty affects health through multiple channels, including limited access to nutrition, healthcare services, sanitation, and education, whereas poor health can reinforce poverty through reduced productivity, indicating a close and reinforcing relationship between poverty and health outcomes [2].

Accordingly, this study addresses the following research question: What is the association between poverty and health outcomes in developing countries? Against this background, and despite extensive evidence that economic growth is associated with improved health outcomes, there remains limited cross‐country evidence directly examining how poverty itself is associated with health outcomes in developing countries. Hence, this study aims to examine the association between poverty and health outcomes in developing countries using a dynamic panel framework. Specifically, the study analyzes data from 63 developing countries using four available observation years within the 2013–2022 and examines whether poverty remains significantly associated with health outcomes after controlling for economic, environmental, and policy‐related factors. A dynamic panel approach is employed to account for persistence in health outcomes and potential endogeneity.

The primary objective of this study is to examine the association between poverty and health outcomes across 63 developing countries using four available observation years within the 2013–2022 period. To address the inherent persistence of health indicators and the potential endogeneity between poverty and health, we employ a dynamic panel data framework utilizing both difference and system generalized method of moments (GMM) estimations. This methodology ensures robust results by controlling for critical economic, educational, environmental, and policy‐related factors. This study contributes to the literature in three distinct ways.

First, instead of relying solely on aggregate indicators, we disaggregate health outcomes into specific dimensions, such as nutrition and medical access, to identify the precise channels through which poverty operates. Second, we utilize poverty gap indicators across multiple international thresholds (e.g., $3.00, $4.20, and $8.30) to provide a more granular assessment of how poverty intensity affects health. Finally, by identifying a “marginal threshold effect,” this research offers novel international evidence that poverty's impact is most pronounced at lower and moderate levels of deprivation. These findings emphasize the need for targeted policy interventions in resource‐constrained contexts rather than relying solely on broad economic growth to improve population health.

The remainder of the article is organized as follows. Section 2 reviews the literature; Section 3 presents the methodology and data; Section 4 discusses the empirical results; and Section 5 concludes with policy implications and limitations.

## 2 Literature Review

A substantial body of literature has examined the relationship between economic conditions and health outcomes, with early studies emphasizing the positive role of income in improving population health. Higher income levels are generally associated with better access to healthcare, improved nutrition, and longer life expectancy [3, 11]. These findings suggest that economic growth can enhance health outcomes by increasing the resources available for health‐related investments.

However, the relationship between economic conditions and health outcomes is not uniform, particularly in developing countries where structural constraints remain significant [2]. In this context, poverty has been identified as a central factor shaping health outcomes. Poverty is closely associated with limited access to healthcare services, inadequate nutrition, poor sanitation, and substandard living conditions, all of which contribute to adverse health outcomes. The persistence of poverty can therefore constrain the extent to which economic improvements translate into better health conditions [2, 15].

Beyond economic and social conditions, a growing body of recent literature from the past 5 years emphasizes the role of environmental and urban factors in shaping health outcomes. Urbanization was historically viewed as a gateway to better health due to the urban advantage, offering superior access to hospitals and treated water. However, recent literature identifies an *urban health paradox* emerging in rapidly developing economies [16]. As cities expand without adequate planning, the benefits of proximity to care are offset by diseases of modernization. Rapid urbanization in developing countries has increased exposure to environmental risks, including air pollution, overcrowding, and extreme heat. Recent global evidence confirms that PM2.5 pollution and rising temperatures significantly increase mortality and morbidity, with the largest burdens falling on low‐income groups [4, 6, 17, 18]. New cross‐country evidence shows that environmental degradation and income inequality jointly erode health gains from economic growth [19, 20, 21]. This includes the double burden of persistent communicable diseases and rising non‐communicable diseases (NCDs) linked to sedentary lifestyles and processed diets. Furthermore, Wang et al. [19] demonstrate that, when environmental quality is considered a critical moderator, ambient air pollution (PM2.5) and the lack of green spaces in megacities impose a health tax on the urban poor, who lack the resources to mitigate these environmental shocks. These findings reinforce the importance of considering poverty alongside environmental and living conditions when analyzing health outcomes.

Despite these advances, most recent studies still treat poverty as a control variable rather than the core explanatory factor in health models. Much research examines income, education, or health spending separately [3, 10, 11] or examines broader dimensions such as environmental risks and sustainability without explicitly centering poverty in the analysis [4, 17, 19]. Few recent cross‐country studies use a unified framework to isolate the independent effect of poverty while accounting for dynamics, endogeneity, and threshold effects. Addressing this gap is particularly important given that poverty affects health through multiple channels, including limited access to healthcare, inadequate nutrition, poor living environments, and reduced capacity to cope with environmental risks [2]. A clearer empirical understanding of the relationship between poverty and health is therefore essential for informing effective policy interventions to improve health outcomes in developing countries. Finally, the selection of control variables in this study is grounded in established macroeconomic and social development theories to ensure the internal validity of the poverty–health relationship and to mitigate omitted variable bias.

First, GDP per capita (GDP) is included to account for the “wealth effect” and the overall level of economic development. According to the “Wealthier is Healthier” thesis [3], higher national income expands the public tax base, facilitating investments in large‐scale sanitary infrastructure and subsidized medical care. By controlling for GDP, we isolate the specific impact of poverty from the general level of economic resources available in the 63 sampled nations, ensuring that our findings reflect the structural constraints of deprivation rather than just a lack of aggregate wealth [2].

Second, secondary education (EDU) is utilized as a proxy for health literacy and human capital. Educational attainment is a critical determinant of “allocative efficiency” in health; more educated individuals are statistically more likely to process health information effectively, adopt preventive behaviors, and navigate complex healthcare systems [1]. Furthermore, higher levels of education in a population are associated with a “virtuous cycle” of improved maternal health and reduced child mortality, making it an essential control for social development [15].

Third, carbon dioxide emissions (CO_2_) are included to capture the environmental externalities of economic growth and urbanization. Environmental quality is a direct input into the health production function; rising CO_2_ levels often serve as a proxy for broader air pollution and climate‐related health risks, such as respiratory illnesses and heat‐related mortality [18]. Including CO_2_ emissions allows this study to account for the “Environmental Health Tax” often paid by populations in rapidly industrializing emerging economies, where economic gains are frequently offset by ecological degradation [19]. Finally, this study uses a composite Health Strategy Index (HSI) rather than relying solely on government health expenditure. This index provides a more holistic representation of a nation's healthcare “readiness” by integrating supply‐side indicators such as food safety, health expenditure, and hygiene/sanitation. As argued by the [4], health outcomes are not merely a function of how much a government spends, but how that investment is translated into physical infrastructure and human resources. By using a composite HSI, we can more accurately measure a health system's buffering capacity against the shocks of poverty and environmental risks.

On the basis of health capital theory and the poverty–health nexus, this study proposes a single hypothesis. Poverty limits the ability of households and communities to secure adequate nutrition, sanitation, healthcare access, and healthy living conditions. In developing countries, these constraints are likely to be more severe because public health systems, social protection mechanisms, and basic infrastructure are often less comprehensive. Although economic growth may improve national resources, poverty can remain a direct structural constraint that prevents health improvements from reaching vulnerable populations. Therefore, after controlling for economic development, education, environmental conditions, price stability, and health‐related strategies, poverty is expected to remain negatively associated with health outcomes.

**H1**. Poverty is negatively and significantly associated with health outcomes in developing countries.

## 3 Methodology

### 3.1 Empirical Framework

This study examines the association between poverty and health outcomes within a macro‐panel framework grounded in health capital theory and the poverty–health relationship [1, 2]. Poverty is considered a key factor associated with population health outcomes, particularly in developing countries where access to healthcare, nutrition, and basic living conditions remains constrained. To examine this relationship, the empirical model is specified as

$$\begin{array}{ccc} HI_{i,t} & = & \beta_{0}+\beta_{1}\mathrm{PO}V_{i,t}+\beta_{2}\mathrm{GD}P_{i,t}+\beta_{3}\mathrm{ED}U_{i,t}+\beta_{4}CO_{2_{i,t}}\\ & & +\beta_{5}\mathrm{CP}I_{i,t}+\beta_{6}HS_{i,t}+\mu_{i}+\varepsilon_{i,t} \end{array}$$

where i denotes country, and t denotes time. HI represents health outcomes, whereas POV captures poverty conditions. The remaining variables control for economic development (GDP), education (EDU), environmental degradation (CO_2_), consumer price index (CPI), and health services [2, 3]. The empirical specification is designed to assess whether poverty remains significantly associated with health outcomes after controlling for other relevant factors. All variables are entered in log form.

To capture persistence in health outcomes, a dynamic specification is also considered:

$$HI_{i,t}=\alpha HI_{i,t-1}+\beta X_{i,t}+\mu_{i}+\varepsilon_{i,t}$$

where $X_{i,t}$ includes all explanatory variables. This dynamic structure reflects the well‐established persistence of health indicators over time [22]. Given the potential endogeneity between health outcomes and explanatory variables, a dynamic panel data approach using the GMM is employed [12, 13]. This approach helps address unobserved heterogeneity and endogeneity while accounting for persistence in health outcomes. Specifically, both difference GMM (DGMM [12]) and system GMM (SGMM [13]) are implemented to address unobserved country‐specific effects, endogeneity of regressors, and dynamic panel bias arising from lagged dependent variables.

However, we acknowledge that the panel structure has a short time dimension (*T* = 4), which may limit the instrument's strength and the estimator's reliability [14]. Therefore, results are interpreted cautiously. To mitigate common GMM‐related concerns, the instrument count is restricted to avoid overfitting [14]. In doing so, instruments are collapsed, and lag depth is limited, and the number of instruments is kept below the number of cross‐sectional units. Model validity is assessed using Arellano–Bond tests for serial correlation (AR(1) and AR(2)) and the Hansen test of over‐identifying restrictions [23]. Only models satisfying the absence of second‐order autocorrelation and acceptable Hansen test results are emphasized. Given the potential overlap among development‐related variables, multicollinearity is assessed using correlation analysis and variance inflation factors [24]. Where high correlations are observed, results are interpreted with caution. (Several limitations should be noted. First, the short time dimension (*T *= 4) may limit the strength of dynamic panel estimation [14]. Second, the use of a composite index may introduce measurement error [25]. Third, cross‐country heterogeneity is not fully captured. Finally, omitted variables, such as institutional quality or urbanization, may influence the results.)

### 3.2 Measurement of Variables—Health Outcome (Dependent Variable)

The dependent variable is a composite health index (HI) capturing multiple dimensions of population health, including life expectancy, mortality rates, nutritional status, and basic healthcare access. To ensure comparability across countries, all indicators are normalized using min–max scaling, a widely used method in composite indicator construction [25]:

$$\;X^{*}=\frac{X-X_{\min}}{X_{\max}-X_{\min}}$$

For negatively oriented indicators (e.g., mortality rates), directional adjustment is applied prior to normalization to ensure consistency. (For negatively oriented indicators (e.g., mortality rates, malnutrition, and crude death rates), directional adjustment is applied using $X^{*}=\frac{X_{\max}-X}{X_{\max}-X_{\min}}$. This ensures that higher values consistently represent better health outcomes across all indicators.) We acknowledge that equal weighting may introduce simplification. However, this approach is commonly adopted in composite index construction when theoretical weighting schemes are not clearly established [25, 26]. The index is therefore interpreted as a proxy for overall health conditions rather than a structural measure. The HI includes life expectancy, chronic disease mortality, nutrition, and health care. The HSI includes the average value of food safety, per capita health expenditure, and basic health conditions. In the HI, life expectancy and medical care are positive indicators, whereas chronic disease mortality, nutrition (the data are malnutrition rate), and mortality are negative indicators. In the HSI, per capita health expenditure and basic health conditions are positive indicators, whereas food security (i.e., food insecurity) is a negative indicator. Therefore, the HI is as follows:

$$\mathrm{Health}\;\mathrm{index}\;\left(\mathrm{HI}\right)=\frac{\mathrm{TIMEI}+\mathrm{MORI}+\mathrm{MEDI}+\mathrm{NUTI}+\mathrm{DEAI}}{5}$$

### 3.3 Measurement of Variables—Independent Variables (Key Explanatory Variable)

The key explanatory variable in this study is poverty, measured using poverty gap indicators at international poverty thresholds [5]. To address potential issues with zero or near‐zero values, log transformations are applied where appropriate, with adjustments to ensure variables are well‐defined and comparable across countries. A small constant is added before the log transformation to handle zero values. Economic development (GDP) is measured by GDP per capita [3], education (EDU) is represented by mean years of schooling [1], environmental conditions (CO_2_) are proxied by carbon emission [9], price stability (CPI) is represented by CPI, and HS is a composite proxy capturing health expenditure, sanitation, and food security [7]. Control variables are included to isolate the association between poverty and health outcomes, rather than to shift the primary focus away from poverty. HS is therefore calculated as

$$\mathrm{Health}\;\mathrm{strategy}\;\mathrm{index}\;\left(\mathrm{HSI}\right)=\frac{\mathrm{FSI}+\mathrm{HEXPI}+\mathrm{HYGI}}{3}$$

The summary of variables, their measurements, and sources is presented in Table 2.

**TABLE 2:** Variables’ measurement and sources.

Proxy	Descriptions	Sources
Poverty level (POV)	Poverty gap at $3.00 a day (2017 PPP) (%) Poverty gap at $4.20 a day (2017 PPP) (%) Poverty gap at $8.30 a day (2017 PPP) (%)	[5]
Health index (HI):		
Life expectancy (TIME)	Life expectancy at birth, total (years)	
Mortality rate (MOR)	Mortality from CVD, cancer, diabetes, or CRD between exact ages 30 and 70 (%)	
Nutrition (NUT)	Prevalence of undernourishment (% of population)	
Death rate (DEA)	Death rate, crude (per 1000 people)	
Medical level (MED)	Immunization, DPT (% of children ages 12–23 months)	
Carbon dioxide (CO_2_)	Carbon dioxide emissions per capita (production) (tons)	[27]
Education level (EDU)	Mean years of schooling (years)	
Gross domestic product (GDP)	GDP per capita (constant $)	[5]
Consumer price index (CPI)	Consumer price index (2010 = 100)	
Health strategy index (HSI):		
Food safety (FS)	Prevalence of severe healthy food insecurity in the population (%)	
Health expenditure (HEXP)	Current per capita health expenditures (constant $)	
Hygiene (HYG)	People using at least basic sanitation services (% of population)	

## 4 Results

This section presents the empirical findings on the association between poverty and health outcomes across developing countries. The analysis examines whether poverty remains significantly associated with health outcomes after controlling for economic, environmental, and policy factors. Table 3 presents the descriptive statistics of the variables used in this study. The average HI across the sample is 72.06, with a relatively wide range from 32.19 to 98.09, indicating substantial variation in health outcomes across countries. This variation suggests that health conditions differ considerably across developing countries, despite their similar income classifications. GDP per capita also shows significant dispersion, with a mean of $3816.45 and a range of $450.51–$13,625.90. Similarly, education levels (EDU) and HS indicators exhibit notable variability, reflecting differences in human capital and policy‐related conditions across countries. Environmental exposure, proxied by CO_2_ emissions, also varies widely, indicating differing levels of environmental risk. Overall, the descriptive statistics highlight considerable heterogeneity in both health outcomes and socioeconomic conditions across the sample, providing an appropriate basis for examining the association between poverty and health outcomes.

**TABLE 3:** Descriptive statistics of variables.

Variable	Obs.	Mean	Std. Dev.	Min	Max
HI	252	72.06	13.29	32.19	98.09
CO_2_	252	2.09	2.84	0.07	15.36
EDU	252	7.28	3.06	1.24	12.82
GDP	252	3816.45	3179.66	450.51	13,625.90
CPI	252	208.54	696.89	103.72	11,076.60
HS	252	54.20	21.33	11.91	97.91

Abbreviations: CPI, consumer price index; EDU, education; GDP, gross domestic product; HI, health index; HS, health strategy.

Table 4 reports the pairwise correlation coefficients among the variables. The HI is positively correlated with GDP (0.64), education (0.51), and HS (0.75), suggesting that countries with better economic and social conditions tend to have higher health outcomes. However, the relatively strong correlations among some explanatory variables, particularly between GDP and HS (0.83), EDU and HS (0.77), and GDP and EDU (0.66), indicate potential multicollinearity. These high correlations suggest that development‐related variables may capture overlapping aspects of socioeconomic conditions.

**TABLE 4:** Correlation coefficient between variables.

	HI	CO_2_	EDU	GDP	CPI	HS
HI	1.00					
CO_2_	0.33	1.00				
EDU	0.51	0.59	1.00			
GDP	0.64	0.57	0.66	1.00		
CPI	0.05	0.05	0.04	0.00	1.00	
HS	0.75	0.54	0.77	0.83	0.03	1.00

Abbreviations: CPI, consumer price index; EDU, education; GDP, gross domestic product; HI, health index; HS, health strategy.

This is important for interpretation, as it implies that the individual effects of these variables may be difficult to disentangle in regression analysis. In contrast, CPI shows very low correlations with other variables, indicating that it may capture distinct macroeconomic conditions. Overall, the correlation results suggest that although economic and policy variables are associated with health outcomes, their effects may not be independent, reinforcing the need to interpret regression results with caution.

The diagnostic tests generally support the use of the dynamic GMM specifications, although the findings are interpreted cautiously given the short time dimension and instrument‐related concerns. The AR(1) and AR(2) tests, along with the Hansen statistic, indicate that the instruments are exogenous and there is no second‐order serial correlation. As shown in Table 5, the lagged health coefficient is positive and highly significant (*p* < 0.01) across all specifications. A key feature of the results is the strong persistence of health outcomes, as indicated by the significant coefficient on the lagged dependent variable. This suggests that health conditions evolve gradually over time, reflecting structural and institutional factors that are not easily altered in the short run [10], indicating the persistence of health outcomes.

**TABLE 5:** Generalized method of moments (GMM) result [DV: lnHI].

	DGMM		SGMM	
	1‐Step	2‐Step	1‐Step	2‐Step
lnHI_(−1)_	0.833(0.000)^***^	0.825(0.000)^***^	1.323(0.000)^***^	1.388(0.000)^***^
lnPOV	−0.019(0.001)^***^	−0.020(0.001)^***^	−0.002(0.090)^*^	−0.001(0.097)^*^
lnHSI	0.033(0.486)	0.045(0.371)	−0.240(0.119)	−0.281(0.036)^**^
lnCO_2_	−0.014(0.320)	−0.016(0.289)	0.073(0.151)	0.072(0.070)^*^
lnEDU	−0.026(0.155)	−0.022(0.090)^*^	−0.074(0.259)	−0.091(0.164)
lnGDP	−0.005(0.777)	−0.004(0.827)	0.028(0.549)	0.043(0.266)
lnCPI	0.012(0.021)^**^	0.010(0.072)^*^	−0.004(0.734)	−0.001(0.915)
	Model criteria			
#Obs	63	63	63	63
#Instruments	59	59	24	24
AR(1)	0.010	0.011	0.082	0.104
AR(2)	0.051	0.152	0.413	0.426
Hansen	0.544	0.544	0.944	0.944

*Note:* The *p* values are reported in parentheses. AR and Hansen refer to the *p* value.

Abbreviations: DGMM, difference GMM; SGMM, system GMM.

*, **, and *** denote the 10%, 5%, and 1% levels of significance, respectively.

Table 5 presents the baseline estimates of the relationship between poverty and health outcomes using dynamic panel models. The results indicate that poverty remains systematically associated with health outcomes across specifications, even after accounting for health condition persistence and potential endogeneity. The coefficient on poverty (lnPOV) is negative and statistically significant in all DGMM specifications at the 1% level, indicating that higher poverty levels are associated with poorer health outcomes. Specifically, the DGMM estimates suggest that a 1% increase in poverty is associated with a decline of approximately 0.019%–0.020% in the HI. This finding highlights the strong association between poverty and health conditions, suggesting that populations experiencing higher poverty levels are associated with poorer access to basic resources and poorer living conditions, which may be linked to poorer health outcomes. In the SGMM, the coefficient on poverty remains negative but is smaller in magnitude. This difference suggests some sensitivity of the estimates to model specification and instrument structure, which is not uncommon in panels with a relatively short time dimension. However, within this dynamic framework, poverty continues to show a stable association with health outcomes, indicating that its role is not subsumed by other control variables. This highlights the importance of poverty as a distinct factor linked to population health, rather than merely reflecting broader economic conditions [2, 15]. These findings are consistent with recent cross‐country studies by [4, 20], which confirm poverty as a generally negative association of health. Similarly, [2, 15] highlight that poverty acts as a structural constraint independent of GDP per capita, supporting our observation that income effects are insignificant after controlling for poverty.

The results for other variables provide additional context but are less consistent. GDP per capita does not exhibit a statistically significant effect across most specifications, suggesting that income alone may not be a sufficient predictor of health outcomes once poverty is taken into account. Similarly, education shows weak, inconsistent effects, suggesting that its impact on health outcomes may depend on broader socioeconomic conditions. Environmental exposure, proxied by CO_2_ emissions, shows limited significance, although the positive coefficients in some specifications suggest that environmental factors may influence health outcomes, particularly in contexts of rapid development. The HS variable displays inconsistent signs across models, which may reflect measurement limitations or potential overlap with broader development indicators. The diagnostic tests indicate that the models are generally well specified. The Arellano–Bond tests indicate significant first‐order serial correlation but no second‐order serial correlation, satisfying the key assumptions of the GMM estimator. The Hansen test results are within acceptable ranges, suggesting that the instruments used are valid. However, given the panel's relatively short time dimension, the results should be interpreted with appropriate caution.

Overall, these findings are consistent with the existing literature, which emphasizes the importance of socioeconomic conditions in determining health outcomes. Studies by [2, 15] highlight the role of poverty and inequality in shaping health disparities, whereas the [7] identifies poverty as a key social determinant of health. In contrast to studies that emphasize income as the primary driver of health improvements [3, 10], the present results suggest that poverty shows a more consistent association with health outcomes. By providing cross‐country evidence from developing countries, this study underscores the importance of addressing poverty as a central component of strategies to improve population health.

Table 6 extends the analysis by disaggregating the HI into its underlying dimensions, allowing for a more detailed assessment of how poverty relates to different aspects of health outcomes. The results in Table 6 provide a more detailed assessment of the association between poverty and specific dimensions of health outcomes, including time‐related health conditions (TIMEI), mortality‐related indicator (MORI), nutrition (NUTI), disease environment indicator (DEAI), and medical access (MEDI). Across these sub‐indicators, poverty generally shows a negative association with health outcomes, though the magnitude and statistical significance vary across dimensions and model specifications. Focusing first on TIMEI, poverty (lnPOV) shows a negative and statistically significant effect in both DGMM and SGMM models, indicating that higher levels of poverty are associated with poorer time‐related health outcomes. This suggests that poverty is associated with differences in access to healthcare services and preventive care.

**TABLE 6:** Two‐step generalized method of moments (GMM) regression results of sub‐indicators of health [DV: lnHI].

	HI = TIMEI		HI = MORI		HI = NUTI		HI = DEAI		HI = MEDI	
	DGMM	SGMM	DGMM	SGMM	DGMM	SGMM	DGMM	SGMM	DGMM	SGMM
lnHI_(−1)_	0.098^**^	0.859^***^	0.317^***^	0.957^***^	1.313^**^	1.177^**^	1.128^***^	1.256^***^	0.396^***^	0.626^**^
	(0.037)	(0.000)	(0.000)	(0.000)	(0.084)	(0.026)	(0.000)	(0.000)	(0.000)	(0.036)
lnPOV	−0.116^**^	−0.017^**^	0.003	−0.008^*^	−0.343^*^	−0.014^*^	−0.005	−0.012^*^	−0.027	0.001
	(0.036)	(0.027)	(0.934)	(0.053)	(0.062)	(0.095)	(0.429)	(0.062)	(0.203)	(0.974)
lnHSI	0.819^***^	−0.040	0.209	0.014	−1.277	0.075	−0.124^***^	−0.157	0.038	0.118
	(0.004)	(0.656)	(0.297)	(0.760)	(0.549)	(0.968)	(0.000)	(0.327)	(0.750)	(0.639)
lnCO_2_	−0.223^**^	0.017	−0.145	0.010	−0.291	0.126	0.007	0.073	−0.016	−0.039
	(0.034)	(0.759)	(0.257)	(0.447)	(0.312)	(0.749)	(0.540)	(0.280)	(0.652)	(0.580)
lnEDU	0.513^**^	0.043	−0.410^*^	0.008	−0.762	0.368	−0.084^**^	−0.104	0.094	0.180
	(0.026)	(0.260)	(0.089)	(0.691)	(0.267)	(0.708)	(0.031)	(0.289)	(0.261)	(0.150)
lnGDP	−0.019	−0.017	0.227^*^	−0.005	1.508	−0.069	0.031	0.012	−0.056	−0.072
	(0.918)	(0.749)	(0.095)	(0.857)	(0.152)	(0.928)	(0.244)	(0.864)	(0.472)	(0.454)
lnCPI	0.144	0.010	0.120	−0.002	0.101	−0.085	−0.002	−0.014	0.032	0.024
	(0.256)	(0.626)	(0.174)	(0.638)	(0.576)	(0.646)	(0.881)	(0.481)	(0.174)	(0.298)
	Model criteria									
#Obs	63	63	63	63	63	63	63	63	63	63
#Instruments	59	24	59	24	59	24	59	24	59	24
AR(1)	0.063	0.077	0.092	0.216	0.071	0.143	0.079	0.082	0.100	0.064
AR(2)	0.251	0.313	0.357	0.891	0.365	0.504	0.377	0.387	0.408	0.289
Hansen	0.676	0.732	0.481	0.192	0.818	0.999	0.575	0.505	0.459	0.647

*Note:* The *p* values are reported in parentheses. AR and Hansen refer to the *p* value.

Abbreviations: DEAI, disease environment indicator; DGMM, difference GMM; HI, health index; MORI, mortality‐related indicator; SGMM, system GMM.

*, **, and *** denote the 10%, 5%, and 1% levels of significance, respectively.

A similar pattern is observed for NUTI, where poverty is negatively associated with nutritional outcomes and is statistically significant in both estimation approaches. This finding is particularly important, as it highlights the strong link between poverty and inadequate nutrition, which is a well‐established determinant of health status [2, 7]. For the MORI, the results are less consistent.

Although poverty is not statistically significant in the DGMM specification, it becomes weakly significant in the SGMM model with a negative coefficient. This suggests that poverty may still influence mortality outcomes, though its effect may be mediated by factors such as healthcare access and demographic conditions. In contrast, for the DEAI, poverty shows a negative, though weakly significant, effect in the SGMM model, indicating that higher poverty levels are associated with less favorable disease‐related conditions. This is consistent with the literature, which emphasizes that poorer populations are more exposed to environmental and epidemiological risks due to inadequate living conditions [15].

The results for medical access (MEDI) show no statistically significant relationship with poverty under either the DGMM or SGMM specifications. This may reflect the fact that access to medical services is influenced not only by individual poverty levels but also by broader institutional and policy factors, such as public healthcare provision and infrastructure. In developing countries, public health interventions may partially offset the direct effects of poverty on medical access, thereby weakening empirical associations. Across all sub‐indicators, the results consistently indicate that poverty is more strongly associated with fundamental health conditions, such as nutrition and time‐related health capacity, than with more institutionally mediated outcomes such as medical access. This pattern suggests that poverty is primarily associated with differences in basic resource conditions, rather than through direct access to formal healthcare systems alone.

The findings are broadly consistent with existing literature on the social determinants of health. Studies by [2] emphasize that poverty influences health through multiple channels, including nutrition, living conditions, and access to basic services. Similarly, Marmot [15] highlights the role of socioeconomic deprivation in shaping health inequalities. Recent global evidence from the [7] further supports the view that poverty remains a central determinant of health disparities, particularly in developing countries. Compared with studies that emphasize income or aggregate economic growth as primary drivers of health outcomes [3, 10], the present results suggest that poverty shows a more consistent association with health outcomes across several dimensions of health. The results indicate that the association between poverty and health is not uniform across dimensions. The relationship appears to be more pronounced for fundamental health conditions, particularly those related to nutrition and basic health capacity. These dimensions are closely related to material living conditions and are more strongly associated with poverty‐related constraints, especially in developing country contexts [2].

In contrast, the association is less consistent for outcomes that are more strongly influenced by institutional or system‐level factors, such as access to medical services. This suggests that the effects of poverty may be partially mitigated in areas where public health systems or policy interventions play a larger role. Such patterns are consistent with the broader literature on the social determinants of health, which emphasizes that structural and institutional factors can moderate the relationship between socioeconomic status and health outcomes [7, 15].

Overall, the disaggregated analysis shows that poverty is primarily associated with differences in basic resource conditions rather than across all dimensions of the health system. This provides a more nuanced understanding of the channels through which poverty is associated with health outcomes.

Table 7 examines whether the association between poverty and health outcomes varies across different poverty thresholds. This analysis provides insight into how the depth and severity of poverty relate to health conditions. This allows for a more nuanced understanding of how the depth and severity of poverty relate to health conditions in developing countries. The results indicate that the relationship between poverty and health is not uniform across thresholds, suggesting that the association between poverty and health outcomes varies with its intensity. At lower poverty thresholds (POV3.00), poverty remains negatively and significantly associated with health outcomes, particularly in the DGMM specification. This suggests that even moderate levels of poverty are associated with poorer health. The persistence of this relationship indicates that relatively small increases in poverty can still have measurable adverse effects on health, likely through constraints on access to basic necessities such as nutrition and primary healthcare services. This finding is consistent with the broader literature, which emphasizes that even marginal deprivation can affect health outcomes [2].

**TABLE 7:** Two‐step generalized method of moments (GMM) regression on sub‐indicators of health [DV: lnHI].

	POV = POV3.00		POV = POV4.20		POV = POV8.30	
	DGMM	SGMM	DGMM	SGMM	DGMM	SGMM
lnHI_(−1)_	0.825^***^	1.388^***^	0.826^***^	1.243^***^	0.865^***^	1.126^***^
	(0.000)	(0.000)	(0.000)	(0.000)	(0.000)	(0.000)
lnPOV	−0.020^***^	−0.001^*^	−0.010^*^	−0.007	−0.006	0.016
	(0.001)	(0.097)	(0.075)	(0.655)	(0.574)	(0.245)
lnHSI	0.045	−0.281^**^	0.070^*^	−0.202	0.032	−0.100
	(0.371)	(0.036)	(0.087)	(0.287)	(0.474)	(0.194)
lnCO_2_	−0.016	0.072^*^	−0.011	0.065^*^	−0.003	0.051^***^
	(0.289)	(0.070)	(0.583)	(0.097)	(0.871)	(0.000)
lnEDU	−0.022^*^	−0.091	−0.060	−0.060	−0.074^**^	−0.051
	(0.090)	(0.164)	(0.128)	(0.321)	(0.032)	(0.152)
lnGDP	−0.004	0.043	0.006	0.019	0.023	0.018
	(0.827)	(0.266)	(0.864)	(0.743)	(0.508)	(0.568)
lnCPI	0.010^*^	−0.001	0.017	0.000	0.018	0.007
	(0.072)	(0.915)	(0.112)	(0.973)	(0.101)	(0.272)
	Model criteria					
#Obs	63	63	63	63	63	63
#Instruments	59	24	59	24	59	24
AR(1)	0.011	0.104	0.019	0.053	0.041	0.043
AR(2)	0.052	0.426	0.093	0.239	0.215	0.218
Hansen	0.544	0.944	0.315	0.659	0.183	0.790

*Note:* The *p* values are reported in parentheses. The model is estimated with a two‐step difference GMM and system GMM. AR and Hansen refer to the *p* value.

Abbreviations: DGMM, difference GMM; POV, poverty; SGMM, system GMM.

*, **, and *** denote the 10%, 5%, and 1% levels of significance, respectively.

Taken together, the results indicate that poverty remains an important factor even after accounting for potential endogeneity and persistence in health outcomes. Crucially, when comparing the three poverty thresholds, we observe a “marginal threshold effect.” The negative impact of poverty is most pronounced at the $3.00 and $4.20 lines, where deprivation is closely associated with reduced access to basic resources like clean water and basic caloric intake. However, as we approach the $8.30 threshold, statistical significance fades (Table 7), suggesting that the association between poverty and health is strongest at lower levels of deprivation. However, at higher income thresholds, health outcomes may be shaped more by institutional quality and environmental factors than by absolute daily income. At intermediate poverty levels (POV4.20), the negative association between poverty and health weakens and becomes less statistically robust. This suggests that as poverty becomes more widespread, its marginal effect on health outcomes may be less pronounced, potentially due to compensatory mechanisms, such as public health interventions or informal support systems. However, the continued negative direction of the coefficients indicates that the underlying relationship between poverty and health remains intact, even if its statistical strength varies across specifications.

At higher poverty thresholds (POV8.30), the relationship between poverty and health becomes statistically insignificant across both estimation methods. This result may reflect threshold effects, in which extremely high levels of poverty are associated with uniformly poor health outcomes, thereby reducing observable variation across countries. In such contexts, additional increases in poverty may not lead to proportionally worse health outcomes because populations are already operating under severe deprivation constraints. This interpretation aligns with the concept of diminishing marginal effects of extreme poverty on observable health variation [15]. Taken together, these results suggest that the impact of poverty on health outcomes is strongest at lower and moderate levels of poverty, where variations in deprivation still translate into meaningful differences in access to health‐related resources. As poverty deepens, however, its marginal effect appears to weaken, possibly due to structural constraints that uniformly limit health outcomes across populations. This highlights the importance of early poverty reduction interventions, as improvements at lower levels of poverty may yield more substantial health benefits.

The results suggest that the relationship between poverty and health is sensitive to the poverty measure used. The association appears more pronounced at lower and moderate poverty thresholds, where variations in deprivation still translate into observable differences in health outcomes. In these contexts, incremental changes in poverty are more likely to be reflected in changes in health conditions, particularly through access to basic services and living standards [2]. At higher poverty thresholds, however, the relationship becomes less distinct. This may reflect a convergence effect, in which countries experiencing widespread poverty exhibit uniformly constrained health outcomes, reducing observable variation. Such patterns are consistent with the notion that extreme deprivation may limit variability in observable outcomes, as populations face broadly similar constraints [15]. This pattern suggests that the marginal association between poverty and health may weaken as poverty deepens, underscoring the importance of accounting for the level of deprivation when interpreting empirical results.

Taken together, the results provide a coherent picture of the relationship between poverty and health outcomes. The baseline estimates establish that poverty remains systematically associated with health outcomes. The disaggregated analysis shows that this relationship is stronger for basic health dimensions linked to material conditions, whereas the threshold analysis indicates that the association's strength varies across levels of deprivation. These findings are consistent with the broader literature, which emphasizes that poverty is a central determinant of health disparities, particularly in developing countries where access to essential resources remains uneven [7]. Overall, the results suggest that poverty operates through multiple, uneven channels, with its influence most evident in areas directly related to basic living conditions.

## 5 Conclusion

This study examines the relationship between poverty and health outcomes in 63 developing countries using four available observation years within the 2013–2022 and applying dynamic GMM estimations. The results generally indicate that poverty is negatively associated with health outcomes. The adverse effects are stronger for basic health dimensions, particularly nutrition, than for institutionally mediated outcomes such as medical access. A threshold pattern is also observed, with the negative association being most pronounced at lower and moderate poverty levels and weaker at higher levels of deprivation. These findings suggest that poverty remains an important structural constraint on health, whereas economic growth alone may not be sufficient to generate broad health improvements. Targeted poverty reduction and investments in basic living conditions therefore remain central to improving population health in developing countries.

The threshold analysis further indicates that the relationship between poverty and health is not uniform. The impact of poverty is more pronounced at lower and moderate levels, whereas its marginal effect weakens at higher levels of deprivation. This suggests that when poverty becomes widespread, health outcomes may converge toward uniformly low levels, reducing observable variation across countries. The overall findings reinforce the importance of poverty as a central factor associated with health outcomes in developing countries. Although other variables, such as income, education, and environmental conditions, play a role, their effects are less consistent than those of poverty. This highlights the need to place greater emphasis on poverty in understanding and addressing health disparities.

The findings of this study have several important policy implications. First, improving health outcomes in developing countries requires more than general economic growth. Although economic development can improve health outcomes, the results suggest that poverty remains a key constraint that must be addressed directly. Policies aimed at reducing poverty, such as income support programs, targeted subsidies, and improved access to basic services, are therefore essential components of health improvement strategies. Second, because poverty has the strongest effects on basic health conditions, interventions in nutrition, food security, clean water, sanitation, and primary healthcare are likely to generate the greatest immediate gains. Third, the variation in results across poverty thresholds indicates that policy effectiveness may depend on the level of deprivation. Interventions at lower and moderate levels of poverty may yield more immediate improvements in health outcomes, whereas in contexts of extreme poverty, broader structural interventions may be required to address deeply entrenched constraints. Finally, the limited and inconsistent effects of some control variables suggest that policies focusing solely on education, income growth, or environmental improvements may not be sufficient in isolation. A more integrated approach that directly addresses poverty alongside these factors is likely to be more effective in improving health outcomes.

This study has several limitations. The short time dimension may restrict the dynamic GMM performance. The composite health and strategy indexes use equal weighting, which involves simplification. Unobserved country heterogeneity and subnational differences are not fully captured. Future research may use longer panels, microdata, and more granular institutional measures to strengthen the findings.

## Author Contributions

**Tajul Ariffin Masron**: methodology, software. **Xiaoxiao Yu**: data curation, investigation, validation formal analysis, supervision, visualization, project administration, resources, writing – original draft. **Tutik Wiryanti Gondo**: visualization, resources. **Dansha Zhang**: writing – review and editing. All authors have read and agreed to the published version of the manuscript.

## Disclosure

The opinions expressed in this article are the authors’ ideas and do not necessarily represent the views of the institutions they belong to.

## Ethics Statement

The authors have nothing to report.

## Conflicts of Interest

The authors declare no conflicts of interest.

## Data Availability

The data that support the findings of this study are available in World Bank at https://databank.worldbank.org/source/world‐development‐indicators. These data were derived from the following resources available in the public domain: World Bank: https://databank.worldbank.org/source/world‐development‐indicators; World Health Organization (WHO): https://www.who.int/data/gho; United Nations Development Programme (UNDP): https://hdr.undp.org/data‐center.
